# On the Use of Electricity in the Treatment of Epilepsy

**Published:** 1878-05

**Authors:** 


On the Use of Electricity in the Treatment of Epilepsy.—That electricity is of some value in the treatment of epilepsy, I have for a long time believed, and while my observattons in this direction do not enable me to assert the exact measure of benefit that we may hope to derive from its use, either alone or in conjunction with approved methods of treatment, they may, perhaps, throw some light on the subject, or, at least, awaken an interest that may lead to a more extended experience as well as to a greater accuracy of observation. I wish to say, first, that in many instances where patients have been submitted to central galvanization—galvanization of the sympathetic, or to general faradization—a profound tendency to drowsiness has been observed.

In some cases sound sleep has for a few moments been induced, with the subject in an upright position while receiving the current through the brain. I recall one patient under treatment by central galvanization who was repeatedly put to sleep within a minute after the beginning of the application.

Accepting the theory that a state of cerebral anæmia predisposes to sleep, it is not very difficult to believe that the feeling of drowsiness that so often follows central galvanization, and even general faradization when specially directed to the cervical ganglion, is due to the direct action of the current on the vasomotor nerves.

It seems hardly necessary at this date to attempt a detailed argument in proof of the very decided action of electricity in any of its manifestations on the arterial circulation.

Among later experiments are those of O. Tschetshott (Abstr. in St. Petrsb. med. Wochenschr., Oct., 1876), and Przewaski (Deutsche med. Wochenshrift, No. 43) who have investigated the action of galvanization of the sympathetic. The latter found that a lowering of the temperature of one side of the face occurs upon faradization of the corresponding ganglia, the decline of temperature ranging from 0.5° to 1.75° Cent., according to the length of the application. In the same way he found that faradization of the ulnar nerve is followed by a decrease from 0.7° to 2.53°

Cent. in temperature in the region between the third and fourth fingers supplied by this nerve. Tschetcchott's results are similar.

I have at this time under treatment a patient who has been under the care of both the late Dr. Peaslee and Dr. Janvrin, and who illustrates, better than any case I ever saw, this control that electricity has over the circulation. For several years this patient has been a great invalid from uterine congestion and displacement, and although now much better of her local troubles, suffers night and day from excessive action of the heart. This activity is altogether functional in character, and in a measure probably dependent on reflex influences, for increase of local pain invariably accelerates the pulse.

It (the pulse) is usually anywhere from 95 to 120, but it frequently rises as high as 160. When at the latter point, an application of general faradization invariably reduces the pulse from 40 to 50, and sometimes 60 beats. When no higher than 125 to the minute, a similar application reduces the number some 30, and when the pulse is beating with its usual frequency, say 95 to a minute, the treatment brings it down some 5 to 10 beats. I have tested this case thoroughly and carefully, and find that no local applications of either the galvanic or faradic currents will give as satisfactory results as general faradization. It is needless to say that the relief following such application is considerable, and the tendency to this unusual excitation seems to be growing less and less.

A case that suggested a certain similarity between the action of bromide of potassium and electricity occurred in the person of a lad, mentioned on page 558 in the second edition of our work on "Medical and Surgical Electricity." The case was one of petit mal, and for two years the attacks occurred from six to ten times a day. In ten-grain doses, the bromide of potassium reduced the paroxysms to two or three in the twenty-four hours. This improvement continued for a month, when, notwithstanding the increased doses of the bromide, the attack gradually increased in frequency. General faradization with galvanization of the sympathetic were then resorted to, and the results that followod were substantially the same as those obtained from the bromide. The epileptic seizures were reduced to from one to three a day, but after a few weeks they returned with their usual frequency. Subsequently, in two other cases of petit mal that came under my notice, the same similarity in the action of the bromide and electricity was noted. Recovery followed in one of these cases. The patient, a girl aged eleven, first observed the attacks in the early part of 1874, and came under my observation in March, 1875. At first the paroxysms occurred but once or twice a week, but in about six months they began to increase in frequency, and for several months before I saw her she was having them as often as once and sometimes three times a day. The attacks were of short duration, lasting not more than one-half to a minute and a half, and although for a time there was perfect unconsciousness, yet the patient, if standing, as was usually the case, never fell, but if engaged in any occupation, immediately resumed it after the attack had passed away. I used at first the bromide of potossium alone, and pushed it until the face was covered with acne. As in other similar cases, she improved very decidedly through several weeks, and then rather quickly relapsed. It is proper to state, however, that this relapse may have been due to a neglect, to which she confessed, of regularity in taking the medicine. I then subjected her to central galvanization and general faradization, alternating the methods and allowing a day to intervene between each application. She improved much less rapidly than under the bromide, but the improvement that followed was retained. At the end of three months she was having but one attack in a period varying from ten days to two weeks, and in just eighteen weeks from the beginning of treatment she had her last attack.

Miss W., aged thirty, came to me November 4, 1875, with the following history: In the early part of 1872 she had her first attack in the night, while asleep, but for a year previous had occasions of being in a dazed condition, with great confusion of memory.

It is as well to state that there had been all along in her case a strong hysterical element that is frequently aggravated by surrounding influences.

The attacks occurred for a while once in about seven weeks, and further on, instead of a single paroxysm, she would have two and sometimes three in the succeeding twenty-four hours. Later still, the attacks became as frequent as once a month, with one or two longer intervals. I learned that Dr. George J. Fisher, of Sing Sing, had formerly been her physician, and, in answer to a letter of inquiry, he informed me that the patient had been under his care for a long time.

He had given her the bromides of potassium and soda (of each ten grains) three times a day. This she had taken for several years, and was still taking when she came under my care. During the month previous she had three attacks, and was feeling certain premonitions, which she described, when I submitted her to the additional treatment of the electricity. I did not feel justified in discontinuing medicine, but in order to give her every chance, substituted for it the following formula of Brown-Sequard:

| | |
|---|---|
| ℞.—Potassæ bicarbonatis, . . . . . | ʒij. |
| Ammonii bromidi, . . . . | ʒvij. |
| Potass. iodidi, . . . . . . | ʒiij. |
| " bromidi, . . . . . . | ℥iij. |
| Infus. calumbæ (British), . . . | Oj. |

M.

S.—Teaspoonful at each meal, and three teaspoonfuls at bedtime.

The patient was exceedingly nervous and despondent, and it was evident that if in no other way electricity migh prove of service, as an adjunct to allay irritability and as a general tonic.

I treated her every other day for three months, alternating central galvanization with general faradization. I then gave her an interval of rest for three months, during which time she had an attack occurring a little more than six months from the last. After a second three months' treatment I allowed another interval of rest, and again treated her for three months. She has not had a second attack, and as eighteen months have passed during which she has had but one seizure, we are hopeful of ultimate results. It is worthy of note that, since the two methods of treatment have been combined, the bromic acne has very considerably lessened, and at times is hardly perceptible.

The last and seemingly the most satisfactory case in its results that I have to relate is as follows: P. M., a peddler, aged thirty-two, entered my office for the purpose of disposing of his wares, March 14, 1877. The man was of respectable appearance and average intelligence, and while conversing with him he had a slight epileptic seizure, and would have fallen from his chair if I had not supported him. When consciousness returned, after what seemed to be a very short period—perhaps not longer than one or two minutes—he seemed to recognize the fact that the attack had been of short duration, and on inquiry gave the following history: He was a native of England, had been in this country two years, and had had the first epileptic attack eighteen months previously. As near as he could remember, the second attack occurred after an interval of six weeks—the third in four weeks; thereafter they gradually increased in frequency, and during the last six months had occurred as often as twice a week. Most of the attacks were light and soon over, as the one that I witnessed, but at least once a month, as he informed me, they were much more severe—the aura being recognizable—with slight lacerations of the tongue.

He had been under no professional care, but had taken a good deal of "salty-tasting medicine," administered to him by some druggists. He recognized the name of bromide of potassium and said he thought that was the medicine.

Upon proposing that he submit himself to my treatment (gratuitously of course) he readily consented, and thinking it to be good and fair opportunity I placed him under central galvanization, with occasional seances of general faradization.

I treated him every day excepting Sundays (when I was out of town) for six weeks. March 16th he had one of his severe attacks. March 22d, one of the lighter seizures. March 31st, another slight attack. April 14th, a third attack of a mild character. On April 20th a paroxysm of the more severe type took place, but evidently considerably modified in intensity and duration.

This was the last severe attack that he had. On May 4th he said that during the morning he thought he had experienced a very slight paroxysm, but it was so transient that he was hardly aware of it. I saw no more of him until the following September, when I accidentally met him on the street. He looked well, said that he had had no more attacks, expressed gratitude for the service rendered, and promised to call at my office, but up to this time he has failed to do so.

In considering these few not altogether unsatisfactory results, a question arises which, in its relation to electricity, has a special significance.

Why do not our therapeutics yield more uniform results? Experience very clearly shows that there is hardly a remedy to the effects of which there is such a varying degree of susceptibility and response as to electricity. To use a strong expression, there are some persons, although perhaps but a small proportion, who were not born to be thus treated. If, while in health, it is given to them, the impression is decidedly unpleasant, or if administered for the relief of pain or nervous irritability, or a general tonic, it fails in its effects, if it does not aggravate the symptoms. On the other hand, there are those upon whom it leaves a delightful impression, and with whom it seems always to agree, and while much does, indeed, depend on the knowledge and adroitness with which the applications are given, this difficulty of foretelling the results that may be expected from the use of the current in given cases is largely the result of inherent differences of susceptibility.

Dr. Meredith Clymer, in some most excellent remarks on the treatment of epilepsy, states that he has never heard of a permanent cure of the disease under the use of the bromides, either alone or in combination.

While we may regard this as an extreme statement, the suggestion, that the best results will follow only when we call to our aid every measure that will tend to increase and develop vital power generally, commends itself to all. It is not alone, therefere, on the theory of a special influence on the nerve-centres, or over the cerebral circulation, that we employ electricity as an adjuvant to the bromides, but also because of its undoubted and powerful constitutional tonic effects. In this, therefore, as in various other forms of central disease, I almost always with central galvanization associate and alternate general faradization. In regard to the frequency of the application I should say, excepting in special cases, the seances, with alterations of the currents, might with advantage be repeated every day. Concerning the length of the applications there is much that might be said, that I can hardly undertake to say here. The universal tendency is to unduly prolong the treatment until secondary exhaustion follows, and more harm results than good. Dr. Lincoln, of Boston, has written an article on this theme which I would commend, at the same time suggesting that we bear in mind the aphorism, "Much too little than a little too much."

I seldom continue the applications of the galvanic current to the brain and sympathetic longer than three minutes, and, as a rule, in from five to ten minutes you accomplish as much by general faradization as is possible. Here as elsewhere, however, individual idiosyncrasies must be studied, and in the details of treatment much is left for the exercise of judgment and experience. In applying galvanic current to the central nerves, I place one electrode—the negative—over the solar plexus and the positive on the top of the head. The sponge which is over the cranial centre, and on which is pressed a large flat metal electrode, should be very large, covering nearly the whole surface of the head.

Small sponges should not be used. They cause too great concentration of current; pain follows, and far less is accomplished. Having the sponges at either pole, large, soft, and applied with firm pressure, and gradually and without interruptions increasing, and in the same way decreasing the current, it is surprising what tension can be borne without the slightest sense of discomfort or unpleasant after-effects.—*New York Medical Record.*

